# Comparative Cytocompatibility and Oxidative Stress Analysis of Green-Synthesized Nano-Silver Fluoride and Silver Diamine Fluoride in Human Gingival Fibroblasts

**DOI:** 10.3390/jfb17040195

**Published:** 2026-04-17

**Authors:** Antonia P. Palankalieva, Iva D. Stoykova, Milen I. Georgiev, Ani B. Belcheva

**Affiliations:** 1 Department of Pediatric Dentistry, Faculty of Dental Medicine, Medical University of Plovdiv, 3 Hristo Botev Blvd., 4000 Plovdiv, Bulgaria; ani.belcheva@mu-plovdiv.bg; 2 Laboratory of Metabolomics, Department of Biotechnology, Institute of Microbiology, Bulgarian Academy of Sciences, 139 Ruski Blvd., 4000 Plovdiv, Bulgaria; ivastoykova_af@mail.bg (I.D.S.); milengeorgiev@gbg.bg (M.I.G.); 3 Department of Plant Cell Biotechnology, Center of Plant Systems Biology and Biotechnology, 4000 Plovdiv, Bulgaria

**Keywords:** dental materials, silver diamine fluoride, nano-silver fluoride, human gingival fibroblasts, nanoparticle toxicity, cytocompatibility

## Abstract

Silver diamine fluoride (SDF) is widely used in pediatric dentistry for caries arrest; however, concerns exist regarding its cytotoxicity. Green-synthesized nano-silver fluoride (NSF) is a potential alternative to SDF, offering antimicrobial efficacy with improved biocompatibility. This study aimed to evaluate the in vitro safety profile of green-synthesized NSF with 5% (*w*/*v*) fluoride using *Camellia sinensis* extract and to compare it with 38% SDF + potassium iodide (KI) formulation in human gingival fibroblasts (HGFs). Eluates of NSF and SDF+KI were tested at serial concentrations of 5%, 1%, 0.1%, 0.01% and 0.005%. Cell viability was assessed after 24, 48, and 72 h using the MTT assay. Additionally, the formation of reactive oxygen species (ROS) in HGFs was detected through fluorescence microscopy. Exposure to 5% SDF+KI resulted in almost complete loss of cell viability at all time points, whereas NSF demonstrated significantly higher viability under the same conditions. Lower concentrations of both materials maintained acceptable biocompatibility. ROS analysis revealed increased oxidative stress in response to 5% SDF+KI, while NSF induced significantly lower ROS levels. NSF exhibited superior biocompatibility compared to SDF+KI, supporting its potential as a safer silver-based material for caries management. Further in vitro and in vivo studies are needed to confirm its clinical safety profile.

## 1. Introduction

Early childhood caries (ECC) is a major global health issue, as it remains a highly prevalent condition with minimal improvement over the past years [1]. Its high incidence inevitably affects the quality of life of children, as well as their parents and caregivers [2]. Early detection of ECC is crucial, as timely diagnosis and intervention can prevent disease progression, reduce the need for extensive restorative treatment, and help avoid associated complications like pain and infection [3]. Moreover, initial caries lesions can be treated non-operatively without the need for rotary instruments, which is beneficial not only from a financial point of view, but also ensures greater patient compliance and comfort [4]. Such minimally invasive methods are very well accepted, especially by young patients as they experience less anxiety compared to traditional operative treatment. All these factors make non-operative treatment a method with great impact in pediatric dentistry [5].

Although fluoride and remineralization products have become a golden standard when treating early caries lesions [6], the recent literature data emphasizes that multiple novel prevention and treatment approaches are being investigated and compared for the management of ECC [7,8].

Silver diamine fluoride (SDF) is a material that is widely used to arrest caries lesions in the dentin [9,10,11,12,13]. Three main properties define its role in arresting caries progression—remineralization, antiproteolytic activity, and antibacterial activity [14].

Its potential efficacy for the treatment of initial enamel lesions has also been explored [15]. However, clinical evidence is still limited compared to the available in vitro data [16,17,18] and further studies are necessary for the validation and support of these treatment strategies.

Regardless of the proven caries-arresting effect, especially in primary dentition, the biggest disadvantage of SDF remains the black staining of teeth, which has limited its use [19]. This staining results from the precipitation of silver particles through the formation of a silver phosphate layer on the carious dentin followed by oxidation [19,20]. To minimize the discoloration, application of potassium iodide (KI) over the SDF was proposed. However, recently it was reported that the improvement in the color after KI application was only temporary and after some time discoloration of the treated teeth still appears [21].

Researchers have sought to develop alternative silver materials that preserve the antimicrobial and caries-arresting efficacy of SDF while overcoming its esthetic drawbacks. In this context, silver nanoparticles (AgNPs) have been proposed as a promising approach for the prevention and management of tooth decay [22]. Due to their nano-scale size they exhibit advanced novel properties which dominate over the bulk-sized silver elements. They have excellent electrical conductivity and chemical stability [23]. With their extremely small size under 100 nm they possess a higher surface area-to-volume ratio, resulting in increased surface energy and a greater number of reactive sites [24]. Recently they have become widely investigated for biomedical applications due to their strong antimicrobial activity, broad-spectrum efficacy, and reduced risk of resistance development [25].

Nano-silver fluoride (NSF) is a relatively new agent, containing AgNPs enriched with sodium fluoride. The first NSF product was created by Targino et al. [26], consisting of silver nitrate reduced chemically using chitosan as a carrier and fluoride as a stabilizer. Throughout the years different approaches to NSF synthesis have been explored, including physical, chemical and biological methods [27]. The conventional physical and chemical methods that produce AgNPs are expensive, toxic, and not eco-friendly. To eliminate these problems researchers have studied natural sources and products for synthesis. The most common ones are plants, because plant phytochemicals show greater reduction and stabilization [28]. *Camellia sinensis* (L.) Kuntze (family Theaceae), green tea, is an excellent option to be used for this purpose in NSF synthesis because it includes over 2000 bioactive compounds [29]. Approximately one third of them are polyphenols, which demonstrate a wide range of biological activities, including antioxidant, anticarcinogenic, anti-inflammatory, thermogenic, probiotic, and antimicrobial properties. Additionally, fluoride—a natural component of green tea—also contributes to its antibacterial potential [30].

In contrast to SDF, NSF formulations exhibit improved stability and do not undergo oxidation when exposed to oxygen, thereby avoiding the characteristic black discoloration associated with SDF application [31]. They retain the antimicrobial and cariostatic benefits of silver compounds—such as the inhibition of *Streptococcus mutans* biofilm and suppression of caries activity and progression [31,32]—which supports their potential as next-generation silver-based materials.

While SDF is widely established as an effective agent for arresting caries lesions, its potential application for enamel lesions in primary teeth remains less clearly defined. Similarly, despite the promising features of NSF, its biological behavior and safety profile in the context of early caries management, particularly in early enamel lesions, have not yet been sufficiently investigated.

In ECC, initial caries lesions are most frequently located on the smooth surfaces of primary teeth in the cervical region, where the topical agents used for treatment may come into close contact with the gingival tissues. Despite this, the potential effects of such materials on the surrounding soft tissues have not been sufficiently explored.

In addition, currently available NSF formulations differ depending on their laboratory synthesis conditions and composition, which may influence their biological behavior. Recent studies have highlighted that the synthesis conditions play a critical role in determining the physicochemical characteristics and biological behavior of nanomaterials [33]. This underlines the need for evaluating each individual formulation under controlled experimental conditions.

In the development of novel materials for biomedical and clinical applications, it is critically important to ensure that they do not exhibit harmful effects when interacting with human tissues. Such materials must undergo comprehensive biocompatibility evaluations to assess their safety profile, including cytotoxicity, oxidative stress, and other biological responses prior to any clinical application [34].

Therefore, the aim of the present study was to conduct an initial biological evaluation focusing on fundamental parameters of cytocompatibility through a comparative assessment of the cytotoxicity of a novel green-synthesized NSF with *C. sinensis* extract and commercially available 38% SDF formulation (Riva Star, SDI Dental Limited, Melbourne, Victoria, Australia) in human gingival fibroblasts (HGFs), as an in vitro model of soft tissue exposure. Reactive oxygen species (ROS) production was subsequently evaluated using confocal fluorescence microscopy to further characterize the cellular response.

## 2. Materials and Methods

### 2.1. Materials

Dulbecco’s Modified Eagle Medium (DMEM) with high glucose 4.5 g/L, 1 mM sodium pyruvate, L-glutamine, and 1.5 g/L sodium bicarbonate (#AL007S, HiMedia, Thane, Maharashtra, India), fetal bovine serum (#F7524, Merck, Rahway, NJ, USA), penicillin/streptomycin/amphotericin B (#A5955, Merck, Rahway, NJ, USA), trypsin–EDTA (#59418C, Gibco, Invitrogen, Waltham, MA, USA), ultra-pure water (#MB1101), 3-(4,5-dimethylthiazol-2-yl)-2,5-diphenyltetrazolium bromide (MTT; #M6494; Sigma-Aldrich, St. Louis, MO 63103, USA), and 2′,7′-dichlorofluoresceindiacetate (DCF-DA; #D6883; Sigma-Aldrich, St. Louis, MO 63103, USA).

### 2.2. Preparation of Test Eluates

Two silver-containing materials were subjected to MTT analysis:

- RS: Commercially available 38% SDF+KI (Riva Star, SDI Dental Limited, Melbourne, Victoria, Australia).
- NSF: Laboratory synthesized NSF using green method with *C. sinensis* extract. The physicochemical characterization of the green-synthesized NSF has been previously reported [35]. The nanoparticles exhibited a predominantly spherical morphology with a surface plasmon resonance at ~400 nm. Dynamic light scattering analysis revealed a monomodal size distribution in the range of 10–100 nm, with an average particle diameter of 65 ± 14 nm. The zeta potential of the synthesized nanoparticles was −36.6 mV, indicating colloidal stability of the nanoparticle suspension. Elemental analysis confirmed the presence of both silver and fluoride within the nanoparticle structure.

The composition of the materials is listed in Table 1.

The eluates were prepared according to the recommendations of ISO 10993-5 [36]. To ensure comparability of the results, the 38% SDF was diluted to a concentration of 5% and mixed with KI in a ratio of 1:2, according to the manufacturer’s recommendations for clinical application.

To avoid the risk of aggregation, both test solutions (RS and NSF) were diluted with ultra-pure water and filtered through a 0.22 µm syringe filter. Then, they were sonicated for 10 min to achieve uniform dissolution of the compounds [37,38]. Finally, each test solution was diluted with ultra-pure water to obtain concentrations of 5%, 1%, 0.1%, 0.01% and 0.005%, which were tested in vitro for 24, 48 and 72 h.

### 2.3. Cell Culture Maintenance

HGF cell culture was purchased from CLS Cell Lines Service GmbH (Eppelheim, Germany) and grown according to the previously described conditions [37,39]. Cells from passages 3 to 7 were used. The HGFs were seeded at a density of 1 × 10^5^ cells per cm^2^ on T75 culture flasks at 37 °C in a humidified incubator with 5% CO_2_. The growth medium was changed every two days, and the cells were sub-cultured when they reached 80% confluence.

### 2.4. Cell Viability Assay

An equal number of HGFs (1 × 10^5^) were cultured with series of experimental concentrations (5%, 1%, 0.1%, 0.01% and 0.005%) from each silver-containing product to analyze cell viability after 24, 48 and 72 h by MTT assay. In addition to the test solutions, control wells with isolated fluoride-free AgNPs were also included to assess background toxicity, as well as control wells containing untreated cells (control) to assess the natural state and viability of the cells without external influences. Briefly, the MTT reagent was added to the wells and incubated for 4 h. Then dimethylsulfoxide (DMSO) at 100 µL per well was added to solubilize the formazan dye. Finally, absorbance at 570 nm wavelength was measured on a microplate reader (Anthos Zenyth 340 multiplate reader, Biochrome, Berlin, Germany).

Cell viability was calculated by the formula:

$$\mathrm{Cell}\;\mathrm{viability}\;\left(\%\right)=\;\frac{\mathrm{OD}\;\mathrm{of}\;\mathrm{treated}\;\mathrm{cells}\;-\;\mathrm{Blank}}{\mathrm{OD}\;\mathrm{of}\;\mathrm{control}\;\mathrm{cells}\;-\;\mathrm{Blank}}\;\times \;100$$

Each experimental condition was performed in quintuplicate and replicated in three separate experiments.

As reported previously [40], cytotoxicity was assessed by determining cell viability following exposure to the tested materials. The half-maximal inhibitory concentration (IC_50_) was intended to be obtained by plotting the percentage of cellular metabolic activity on the Y-axis against the corresponding concentrations of each silver material formulation on the X-axis. Non-linear regression analysis was planned using GraphPad Prism software (version 8.1.0, GraphPad Software Inc., San Diego, CA, USA). The applicability of IC_50_ calculation depended on the obtained dose–response data.

Additionally, the changes in cell morphology after 24 h of treatment with each tested group were examined by light microscopy at 40× magnification and 20 μm scale.

### 2.5. Fluorescence Microscopy for ROS Formation

To determine the levels of ROS generation, the HGF cells were seeded at a density of 3.1 × 10^5^ cells/well in μ-slide 8-well chambers (Ibidi GmbH, Gräfelfing, Germany) with optical bottoms [41]. At the 48th hour of seeding, the cells were pre-treated with NSF and RS at concentrations of 5%, 0.01%, and 0.005%, and AgNPs only at a concentration of 100% for 24 h. Next, following two PBS washes, 20μM of the 2′,7′-dichlorofluoresceindiacetate (DCF-DA) solution was added to each well. Control cells were also included under the same conditions without treatment with the target therapies. Fluorescence was detected after 30 min under a Fluorescent DMi8 inverted microscope from Leica (Wetzlar, Germany) with an FITC filter. Leica LAS X software v.1.4.5 27713 (Wetzlar, Germany) was used for image assessment. Normalized relative fluorescence units (RFU) were calculated relative to the negative control values, represented as the normalized pixel intensity using ImageJ software (v.1.53t, ImageJ, Madison, WI, USA).

### 2.6. Statistical Analysis

Statistical analysis was performed using SigmaPlot v.11.0 (Systat Software GmbH, Erkrath, Germany). The viability assay was conducted in three independent biological experiments, and the data were represented as mean ± standard error of the mean (SEM). The Shapiro–Wilk test was used to assess the normality of data distribution. For normally distributed data, statistical significance was determined by one-way analysis of variance (ANOVA), followed by Bonferroni’s post hoc test. When the data did not follow a normal distribution, comparisons were performed using the Mann–Whitney test, followed by Dunn’s post hoc test.

Representative fluorescent microphotographs of the HGFs were selected from at least ten technical replicates per group.

## 3. Results

### 3.1. Biological Effects of NSF and RS on Cellular Morphology and Cytotoxicity

**Figure 1:**
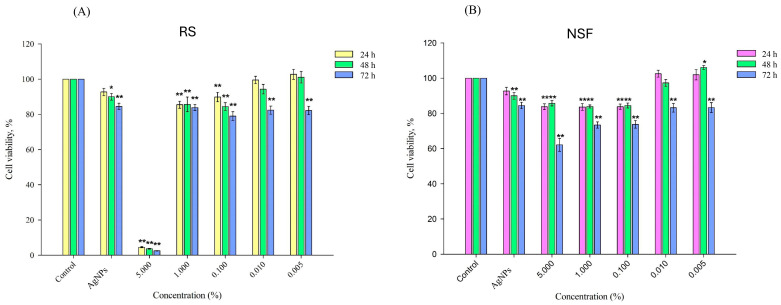
Concentration and time-dependent effect of RS (**A**) and NSF (**B**) on HGFs. *—*p* < 0.05; **—*p* < 0.01.

The mean values for each concentration of each tested silver-containing product are represented in Table 2 and the data is visually shown in Figure 1.

The analysis of the results showed that the control group with untreated cells retained 100% cell viability in all time periods of the experiment. Treatment with fluoride-free AgNPs demonstrated the reduced viability of HGF cells in a time-dependent manner—from 92.67% at 24 h to 84.5% at 72 h, indicating minimal cytotoxicity. In contrast, the cells treated with RS exhibited a marked viability decrease at a 5% concentration of the tested material at 24 h (4.54%), with the effect becoming even more pronounced at 48 h (3.63%) and 72 h (2.51%). The lower concentrations of the product (1%, 0.1%, 0.01%, and 0.005%) showed significantly weaker cytotoxicity, recorded in all time points, an indicator of which was the increase in the percentage of cell viability.

Cells exposed to laboratory-synthesized NSF demonstrated consistently high percentages of cell viability at all concentrations. At the highest concentration (5%), NSF exhibited significantly greater biocompatibility than RS, with cell viability values of 83.79% at 24 h and 85.72% at 48 h. The lowest HGF viability in the NSF group was observed at the 5% concentration after 72 h of exposure (62.11%), remaining, however, substantially higher than the recorded value for RS at the same concentration and time point. The lower concentrations of NSF (1%, 0.1%, 0.01%, and 0.005%) maintained high cell viability, demonstrating comparable or improved biocompatibility to the same concentrations of RS at all time points. Notably, no concentration of the NSF formulation reached the 50% viability threshold at any time point. Therefore, calculation of IC_50_ was not applicable, as the experimental data did not reach the inhibitory range required for reliable model fitting. These findings indicate that NSF did not exert any significant cytotoxic effects under the tested conditions and support its favorable biocompatibility profile. For the RS group, although a marked reduction in cell viability was observed at the highest concentration of 5%, a sharp transition in response occurred between 5% and 1%, with no intermediate data points corresponding to approximately 50% inhibition. As a result, the dose–response relationship did not exhibit a classical sigmoidal profile, and non-linear regression analysis would yield unstable and potentially biased estimates. Therefore, IC_50_ values were not reported, and cytotoxicity was interpreted based on observed viability trends and statistical comparisons. This approach is consistent with established recommendations that IC_50_ values should only be reported when a valid dose–response relationship is achieved [42].

**Figure 2:**
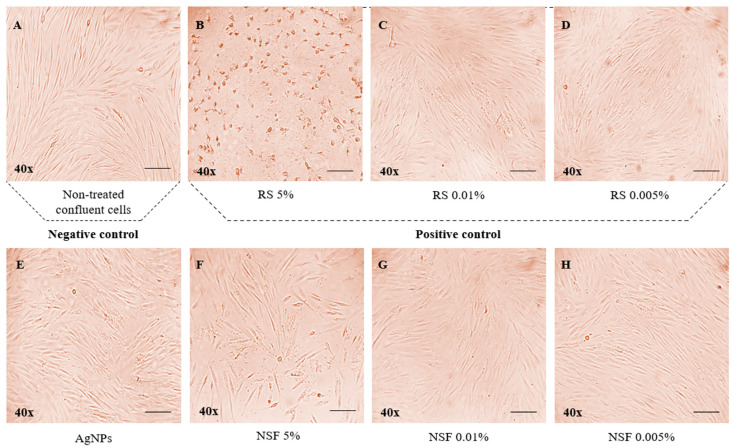
Cell morphology after 24 h of treatment with each tested group was examined by light microscopy at 40× magnification and 20 μm scale in HGF cells. (**A**) Untreated HGFs; (**B**) HGFs treated with 5% RS; (**C**) HGFs treated with 0.01% RS; (**D**) HGFs treated with 0.005% RS; (**E**) HGFs treated with AgNPs; (**F**) HGFs treated with 5% NSF; (**G**) HGFs treated with 0.01% NSF; (**H**) HGFs treated with 0.005% NSF.

As for morphological changes (Figure 2), no significant ones were observed in the cell culture with treatments at the low concentrations of NSF (Figure 2G,H) and RS at 0.01%, and 0.005% (Figure 2C,D), as well as with AgNPs alone (Figure 2E), compared to the untreated control cells (Figure 2A). However, when exposed to the 5% concentration of RS (Figure 2B), HGFs showed significant changes and characteristics of cell death, including cell shrinkage, few cell extensions, increased floating cells, and an accumulation of clusters in the cytoplasm. This corresponds to the low percentage of cell viability detected by the MTT assay. As for the 5% NSF, a mixed cellular response was observed (Figure 2F). Although some cells demonstrated signs of cell death, a considerable proportion retained the characteristics of vitality, which aligns with the obtained results from the MTT analysis.

### 3.2. Detection of ROS Levels upon Treatment with NSF and RS in HGFs

The reduction in ROS levels is considered an indicator for antioxidant activity on the toxic hydrogen peroxide generated by the cells.

**Figure 3:**
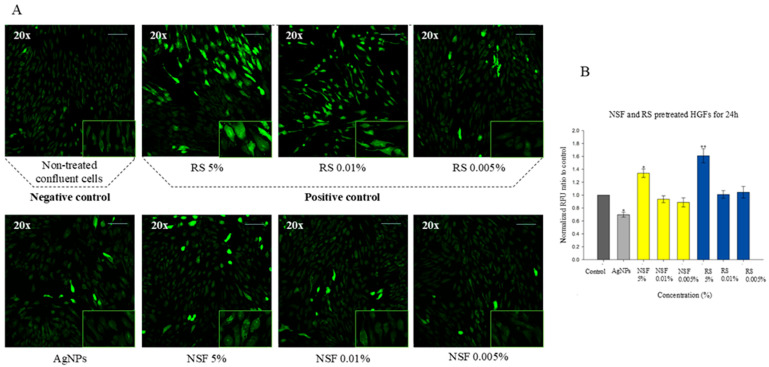
Evaluation of oxidative stress induced by RS and NSF in HGFs. (**A**) Representative fluorescence images demonstrating intracellular ROS accumulation after 24 h exposure; (**B**) Quantitative measurement of ROS production (RFU). *—*p* < 0.05; **—*p* < 0.01.

To investigate the potential role of oxidative stress induced by NSF and RS, ROS generation was measured and compared to untreated cells. The changes in cell morphology observed as an indication of oxidative stress are shown in Figure 3A, whereas Figure 3B illustrates the corresponding quantitative assessment of ROS production (RFU) in HGFs after 24 h of treatment with the respective materials and concentrations. Untreated cells exhibited minimal fluorescence, indicating low baseline ROS levels. Exposure to 5% RS resulted in markedly increased green fluorescence, suggesting higher oxidative stress. Lower RS concentrations (0.01% and 0.005%) demonstrated reduced fluorescence intensity, suggesting a concentration-dependent effect. In contrast, NSF-treated groups showed lower fluorescence intensity across all concentrations compared to RS. At 5%, NSF induced a moderate increase in ROS levels (1.34 RFU), which remained lower than that observed for 5% RS (1.61 RFU), indicating comparatively improved cytocompatibility. Interestingly, the lower concentrations of NSF (0.01% and 0.005%) demonstrated ROS levels below those of the untreated control group, suggesting a potential protective effect on HGF cells. Treatment with isolated AgNPs without added fluorides also showed decreased ROS levels compared to the untreated control, as well as compared to all tested NSF and RS groups.

## 4. Discussion

Cytotoxicity assays are well-established methods for assessing the toxicity of various substances to biological tissues. They represent the gold standard for preliminary safety evaluation and screening, while also having a rapid and easy-to-perform protocol [43]. In the MTT assay, tetrazolium dye is reduced to formazan by the activity of mitochondrial enzymes. The amount of formazan formed is directly proportional to the number of cells that have retained their viability after treatment with the test material [44].

According to ISO standards, a material can be considered cytotoxic when the percentage of cell viability decreases by more than 30% after performing an MTT test [45].

Extensive studies on the cytotoxicity of silver compounds against various cell types are available in the literature—including human peripheral lymphocytes [46], pulp stem cells [47,48], and mice pulp cells [49]. HGFs are a preferred cell model for assessing the cytotoxicity of various dental materials [50]. They play a key role in maintaining the structural integrity of gingival tissues; producing collagen, elastin, and glycosaminoglycans; participating in inflammatory reactions within the gingiva; interacting with various immune cells; and mediating the release of cytokines and growth factors [51,52]. As the clinical application of silver materials for desensitizing and caries-arresting purposes is related to being in close proximity and possible contact with gingiva, this led to the choice of HGFs as the cell model to assess the cytotoxicity of the investigated materials in the present study.

Studies by Fancher et al. [53], Ho et al. [54], and Shijia et al. [55] demonstrated that 38% SDF was highly cytotoxic to HGFs, leading to their almost complete destruction. Also, recent research by Mebin et al. [56] revealed that SDF induces pronounced dose-dependent cytotoxic effects in HGFs, with oxidative stress playing a central role in the observed cellular damage. However, these analyses did not consider whether there was a change in the toxicity when KI was applied to minimize the black staining of teeth after SDF application.

Up to the time of this study, no literature data was found on the cytotoxicity on HGFs of eluate from the commercial product Riva Star, containing SDF and KI, mixed in a ratio of 1:2 according to the manufacturer’s recommendations. Garcia-Bernal et al. [36] performed a similar in vitro study, investigating the cytotoxic effects of the individual Riva Star components (Step 1 and Step 2), as well as their combination (Step 1 + Step 2) using mesenchymal stromal cells. Their findings demonstrated that the combination of SDF+KI has improved in vitro cytocompatibility compared to SDF alone.

The results of the present MTT assay showed that exposure of HGFs to a 5% eluate of SDF and KI in a ratio equal to 1:2 results in almost complete cell death. Concentrations of 1% and lower, however, can be considered biocompatible and non-toxic for cell models. Additional studies are needed to confirm these results and better understand the mechanism of cytotoxic action.

Although the remineralizing and antibacterial properties of 38% SDF+KI have been extensively documented and widely recognized, its potential adverse effect on soft tissues should not be overlooked. Direct contact with gingival tissue may result in localized damage, necessitating careful application in clinical settings. To minimize the risk of potential side effects, 38% SDF+KI should always be applied after adequate isolation of soft tissues with a gingival barrier and protection of the adjacent areas with petroleum jelly, in strict accordance with the manufacturer’s instructions for use.

In recent years, interest in nanotechnology in dentistry has been growing. For a new product to be introduced into clinical practice, it must undergo a series of biotolerance and toxicity tests. In this regard, more studies are aimed at assessing the safety of nanomaterials, and particularly AgNPs [57,58,59].

According to the available literature, green-synthesized AgNPs show selective toxicity—they are toxic to oral cancer cells while largely preserving the vitality of normal cells. This was demonstrated by Ghabban et al. [60], who produced nanoparticles using extracts from *Astragalus spinosus* (Forssk.) Muschl. as a stabilizing agent. Similarly, Khorrami et al. [61] synthesized nano-silver particles using green walnut bark extract. They observed it exhibited low toxicity to L-929 fibroblast cells, while cancer cells from the MCF-7 line showed reduced viability after treatment. Although these studies have investigated the cytotoxicity of AgNPs synthesized using plant extracts other than *C. sinensis* and without the incorporation of fluoride compounds, they nevertheless provide important evidence supporting the biocompatibility and safety of such nanomaterials for potential application in the oral cavity.

In this context, a study by Yin et al. [62] compared the cytotoxic effect of NSF stabilized with chitosan and reduced with epigallocatechin gallate to silver nitrate on HGFs. The results showed that NSF has significantly lower toxicity and is a safer alternative to silver nitrate. To the best of our knowledge, this is the only study that conceptually approaches the present one.

Building on this limited evidence base, the present study evaluates for the first time the cytotoxicity of a laboratory-synthesized NSF obtained by a green method with green tea extract employing MTT and ROS assays. In contrast to SDF+KI, the green-synthesized NSF demonstrated significantly improved biocompatibility at equivalent concentrations, particularly at 5%, where cell viability remained substantially higher. From a biomaterials perspective, NSF represents a complex material, in which antibacterial and remineralization properties and biological compatibility are united in a single formulation. Moreover, its nano-scale dimensions enable a high surface-to-volume ratio and improved interaction with the dental tissues, while minimizing possible acute cytotoxic effects on the surrounding soft tissues. Collectively, these characteristics make NSF a promising alternative to conventional silver-based materials.

Nevertheless, the limitations of the study should be taken into consideration. While the present study provides an evaluation of the biological safety of the investigated materials based on cytotoxicity and oxidative stress assays, the use of a two-dimensional in vitro model with a single cell type does not fully replicate the complexity of the oral microenvironment. Importantly, the study was designed as a first-line biological assessment, as cytotoxicity and ROS generation represent fundamental and widely accepted indicators of cellular response and material safety in the early stages of biomaterial evaluation. These parameters provide essential baseline information prior to more advanced investigations. Further analyses using additional cell models and more complex approaches, such as antibacterial, biofilm, and inflammatory response analyses, are planned in future work to confirm the safety of NSF prior to its potential clinical use as a caries-arresting agent.

## 5. Conclusions

The present study demonstrated that the investigated green-synthesized NSF exhibited lower cytotoxicity and reduced oxidative stress in HGFs compared to SDF+KI under the tested conditions. These findings suggest a more favorable in vitro biocompatibility profile of this specific NSF formulation. However, given the preliminary nature of the experimental model, these results should be interpreted with caution. Further studies, including more complex biological models and in vivo investigations, are required to confirm the safety and potential clinical relevance of these findings.

## Figures and Tables

**Table 1:** Composition of the silver-containing products, subjected to MTT analysis.

Material	Composition
38% SDF+KI (Riva Star capsule kit, SDI Dental Limited, Melbourne, Victoria, Australia)	Step 1 (Silver capsule): Ag, F, NH_3_ Step 2 (Green capsule): saturated KI
Green-synthesized NSF (laboratory-prepared)	AgNO_3_, K_2_CO_3_, NaF, aqueous extract of *C. sinensis*

**Table 2:** Descriptive statistics (mean ± SEM) for HGF viability after treatment with different concentrations of RS and NSF at 24, 48, and 72 h. *—*p* < 0.05; **—*p* < 0.01.

		24 h		48 h		72 h	
	Concentration (%)	Mean (%)	SEM	Mean (%)	SEM	Mean (%)	SEM
Control		100		100		100	
AgNPs		92.67	2.1	89.99 *	1.9	84.50 **	1.8
RS	5	4.54 **	0.3	3.63 **	0.2	2.51 **	0.1
	1	85.46 **	2.0	85.66 **	4.1	83.89 **	1.7
	0.1	89.80 **	2.7	84.42 **	2.2	79.00 **	2.5
	0.01	99.50	2.1	94.28	2.7	82.37 **	2.3
	0.005	102.74	2.9	101.08	3.3	82.18 **	2.3
Control		100		100		100	
AgNPs		92.67	2.1	89.99 **	1.9	84.50 **	1.8
NSF	5	83.79 **	1.6	85.72 **	1.5	62.11 **	3.7
	1	83.63 **	2.0	83.91 **	0.9	73.36 **	1.8
	0.1	83.81	1.5	84.45	1.4	73.76 **	2.2
	0.01	102.55	2.0	97.33	1.9	83.19	2.5
	0.005	101.95	2.9	106.04 *	1.1	83.30 **	2.8

## Data Availability

The data supporting the findings of this study are available from the corresponding author upon reasonable request.

## Abbreviations

The following abbreviations are used in this manuscript:

ECC	Early childhood caries
SDF	silver diamine fluoride
KI	potassium iodide
NSF	nano-silver fluoride
HGF	human gingival fibroblasts
AgNPs	silver nanoparticles
MTT	3-(4,5-dimethylthiazol-2-yl)-2,5-diphenyltetrazolium bromide
ROS	reactive oxygen species
DMEM	Dulbecco’s Modified Eagle Medium
DMSO	dimethylsulfoxide
DCF-DA	2′,7′-dichlorofluoresceindiacetate
IC_50_	half-maximal inhibitory concentration
RFU	relative fluorescence intensity
SEM	standard error of the mean
ANOVA	analysis of variance
